# Impact of surgical site infection on patients’ outcome after fixation of tibial plateau fractures: a retrospective multicenter study

**DOI:** 10.1186/s12891-021-04402-6

**Published:** 2021-06-09

**Authors:** Ralf Henkelmann, Richard Glaab, Meinhard Mende, Christopher Ull, Philipp-Johannes Braun, Christoph Katthagen, Tobias J Gensior, Karl-Heinz Frosch, Pierre Hepp, Committee TRAUMA of the AGA-Society for Arthroscopy and Joint Surgery

**Affiliations:** 1 Department of Orthopedics, Trauma and Plastic Surgery, University of Leipzig, Liebigstraße 20, 04103 Leipzig, Germany; 2 Department of Traumatology, Cantonal Hospital Aarau, Tellstrasse 25, 5001 Aarau, Switzerland; 3 University of Leipzig, Centre for Clinical Trials, Härtlestr. 16/18, 04107 Leipzig, Germany; 4 Department of General and Trauma Surgery, BG University Hospital Bergmannsheil, Bürkle de la Camp-Platz 1, 44789 Bochum, Germany; 5 Department of Trauma and Orthopaedic Surgery, BG Hospital Unfallkrankenhaus Berlin gGmbH, Warener Str. 7, 12683 Berlin, Germany; 6 Department of Trauma, Hand and Reconstructive Surgery, University Hospital Münster, Waldeyerstraße 1, 48149 Münster, Germany; 7 Clinic for Arthroscopic Surgery, Sports Traumatology and Sports Medicine, BG Clinic, Großenbaumer Allee 250, 47249 Duisburg, Germany; 8 Orthopädische Gemeinschaftspraxis Neuss, Breite Str. 96, 41460 Neuss, Germany; 9 Clinic of Trauma, Hand and Reconstructive Surgery, University Medical Center Hamburg-Eppendorf, Martinistraße 52, 20251 Hamburg, Germany; 10 Department of Trauma and Reconstructive Surgery, Division of Knee and Shoulder Surgery, Sports Traumatology, Asklepios Klinik St. Georg, Lohmühlenstraße 5, 20099 Hamburg, Germany

**Keywords:** Outcome tibial plateau fracture, surgical site infection, Knee Injury and Osteoarthritis Outcome Score, Lysholm knee scoring scale

## Abstract

**Background:**

Surgical site infection (SSI) occurs in 3–10 % of patients with surgically treated tibial plateau fractures. This study aimed to evaluate the impact of SSI on patients’ outcome after fixation of tibial plateau fractures.

**Methods:**

We conducted a retrospective multicenter study in seven participating level I trauma centers between January 2005 and December 2014. All participating centers followed up with patients with SSI. In addition, three centers followed up with patients without SSI as a reference group. Descriptive data and follow-up data with patient-reported outcome scores (Knee Injury and Osteoarthritis Outcome Score [KOOS] and Lysholm knee scoring scale score) were evaluated.

**Results:**

In summary, 287 patients (41 with SSI and 246 without SSI; average 50.7 years) with an average follow-up of 75.9 ± 35.9 months were included in this study. Patients with SSI had a significantly poorer overall KOOS (KOOS5) (48.7 ± 23.2 versus [vs.] 71.5 ± 23.5; p < 0.001) and Lysholm knee scoring scale score (51.4 ± 24.0 vs. 71.4 ± 23.5; p < 0.001) than patients without SSI. This significant difference was also evident in the KOOS subscores for pain, symptoms, activities of daily living (ADL), and quality of life (QoL). SSI remained an important factor in multivariable models after adjusting for potential confounders. Clinically relevant differences in the KOOS5 and KOOS subscores for symptoms, pain, and ADL were found between those with SSI and without SSI even after adjustment. Furthermore, the number of previous diseases, Arbeitsgemeinschaft für Osteosynthesefragen Foundation (AO) C fractures, and compartment syndrome were found to be additional factors related to poor outcome.

**Conclusions:**

Compared to previous studies, validated patient-reported outcome scores demonstrated that the impact of SSI in patients with surgically treated tibial plateau fractures is dramatic, in terms of not only pain and symptoms but also in ADL and QoL, compared to that in patients without SSI.

## Background

The goal of the surgical treatment of tibial plateau fractures (TPFs) is to achieve the best level of mobility and quality of life (QoL) postoperatively. However, the postoperative outcome depends on many factors such as the patient’s general health, injury severity with fracture morphology and soft tissue injury, the surgical approach with quality of fracture reduction, and the occurrence of postoperative complications including posttraumatic arthrosis [1–9]. The initial surgical treatment strategy is determined by the fracture morphology, soft tissue damage, concomitant injuries, and patient’s general condition. In the early postoperative phase, surgical site infection (SSI) is the most feared complication. Average SSI rates of ≥ 4.5 % have been described [10–12]. SSIs often lead to multiple revision surgeries, delayed recovery, and transient or permanent loss of function in the affected region [13, 14]. Several independent risk factors for SSI have already been identified. Some of these factors may be influenced, offering the potential to reduce the rate of SSI in the future [11, 15].

In addition to these findings, little is known to date about the specific outcome after SSI. A systematic review showed that only 44 % of patients have a satisfactory outcome after a deep SSI [16]. However, robust data with validated scores on this issue are still missing. Thus far, the focus of the most studies is to evaluate the general outcome of TPFs [5, 17–19].

Therefore, the present study aimed to evaluate the impact of SSI on outcome after operative treatment of TPF based on patient-reported outcome scores.

## Methods

### Study design and setting

We performed a retrospective multicenter study at seven level I trauma centers in Germany and Switzerland. The study was approved by the leading ethics committee of the University of Leipzig (Reference number: 098/15-ff) and all corresponding ethics committees of the participating centers. An analysis of epidemiologic data from this collective to identify independent risk factors has been previously published [15]. As a next step and for further analysis, all patients with an SSI were invited for follow-up at all seven study centers. Furthermore, in three of the seven study centers, patients without an SSI were additionally invited to undergo follow-up as the reference group.

### Patients and eligibility criteria

All patients who had had surgical treated TPFs at one of the hospitals from January 2005 through December 2014 were identified by querying the hospitals’ databases with the International Classification of Disease code for proximal tibia fractures. To avoid inclusion of patients who were improperly coded, those operated on at another hospital, and those who did not meet our inclusion and exclusion criteria, we screened every patient manually.

Inclusion criteria were patient age > 18 years, primary treatment undergone in one of the participating hospitals, and proximal tibia fractures according to the Arbeitsgemeinschaft für Osteosynthesefragen Foundation/Orthopaedic Trauma Association (AO/OTA) 41 B or C [20]. Exclusion criteria were previous surgery at the fracture site performed at another hospital, AO 41 A fractures, and pathological fractures.

### Variables, data sources, and measurements

All variables to be recorded were specified in advance and communicated to all participating centers using a pre-prepared spreadsheet. In addition to the standard parameters (age, sex, etc.), comorbidities were categorized into four groups according to the number of comorbidities: none, 1–3 comorbidities, 4–5 comorbidities, ≥ 6 comorbidities. Comorbidities included predefined diseases such as diabetes mellitus or hypertension; details have been published elsewhere [15]. The variables diabetes mellitus, nicotine abuse, alcohol/drug abuse as well as immunosuppressive drugs were listed separately at the nominal scale level.

Accompanying injuries to the affected knee joint were classified as none, not relevant (abrasions and soft tissue injuries classified as grade 1 according to the Gustillo and Anderson classification), and relevant (further fractures of the affected extremity and compartment syndrome). Other concomitant injuries were categorized as none, not relevant (hematoma, abrasions, and craniocerebral trauma grade 1), and relevant (fracture to other body region and craniocerebral trauma > 1 grade). Furthermore, patients with an injury severity score (ISS) > 16 were classified as having polytrauma [21].

The fracture morphology was classified according to the AO/OTA classification [20]. Furthermore, the variables open fracture and compartment syndrome were considered.

SSI was recorded according to the definition proposed by the current protocol of the National Healthcare Safety Network, Centers for Disease Control and Prevention. These definitions are used in the German guideline of the Robert Koch Institute and by the World Health Organization [22].

Outcome was measured using two different patient-reported outcome (PRO) scores: the overall Knee Injury and Osteoarthritis Outcome Score (KOOS5) with its corresponding subscores (symptoms, pain, activities of daily living [ADL], function/sports and recreational activities [Sport/Rec], and QoL) and the Lysholm knee scoring scale score [23–25]. For the KOOS5 score (maximum 100), a lower score represented more symptoms or pain, greater difficulty performing ADLs and Sport/Rec, and poorer QoL. This rating is also valid for the Lysholm knee scoring scale (maximum 100).

A minimum follow-up was determined at 12 months postoperatively. The follow-up examination of the patient group with SSI was performed at all participating centers. All patients with SSI were contacted and followed up with by telephone or mail. Additional follow-up examination of the reference group with patients without SSI was conducted at three centers.

### Bias

Because of the retrospective study design and the large number of study centers, it is possible that patients with SSI may not have returned to one of the study centers for further treatment. Thus, the number of SSIs may be higher, and the influence of a higher number of patients with SSIs may have an impact on the results.

### Statistical methods

The study cohort was characterized by standard statistics: mean value (standard deviation) for continuous data and number (percent) for categorical data. Patient groups with and without infection were compared using t-test for continuous measurements and chi-square test without correction for cross tables.

We added analyses using general linear models with three objectives: First, we reduced bias by adjusting for confounders by including imbalanced variables in multiple models. Second, these models were used to estimate the effects of known clinical parameters in multiple settings. Third, we separated important variables from negligible variables by simplifying the models. We started with a full model that included SSI, age, sex, body weight, preconditions, smoking and substance abuse, AO classification, and presence of polytrauma, compartment syndrome, concomitant injury, and external fixation. This model was simplified by stepwise backward variable rejection to minimize the Akaike information criterion. The final models were calculated by forcing the remaining variables into the linear model.

All tests were performed two-sided to the significance level α = 0.05. The analyses were performed with SPSS Statistics version 26 (IBM Corp.) and R software (R Core Team).

## Results

### Participants

Between January 2005 and December 2014, 2106 patients were included in the overall study, including 94 patients with SSI, which corresponds to a rate of 4.5 %.

**Fig. 1:**
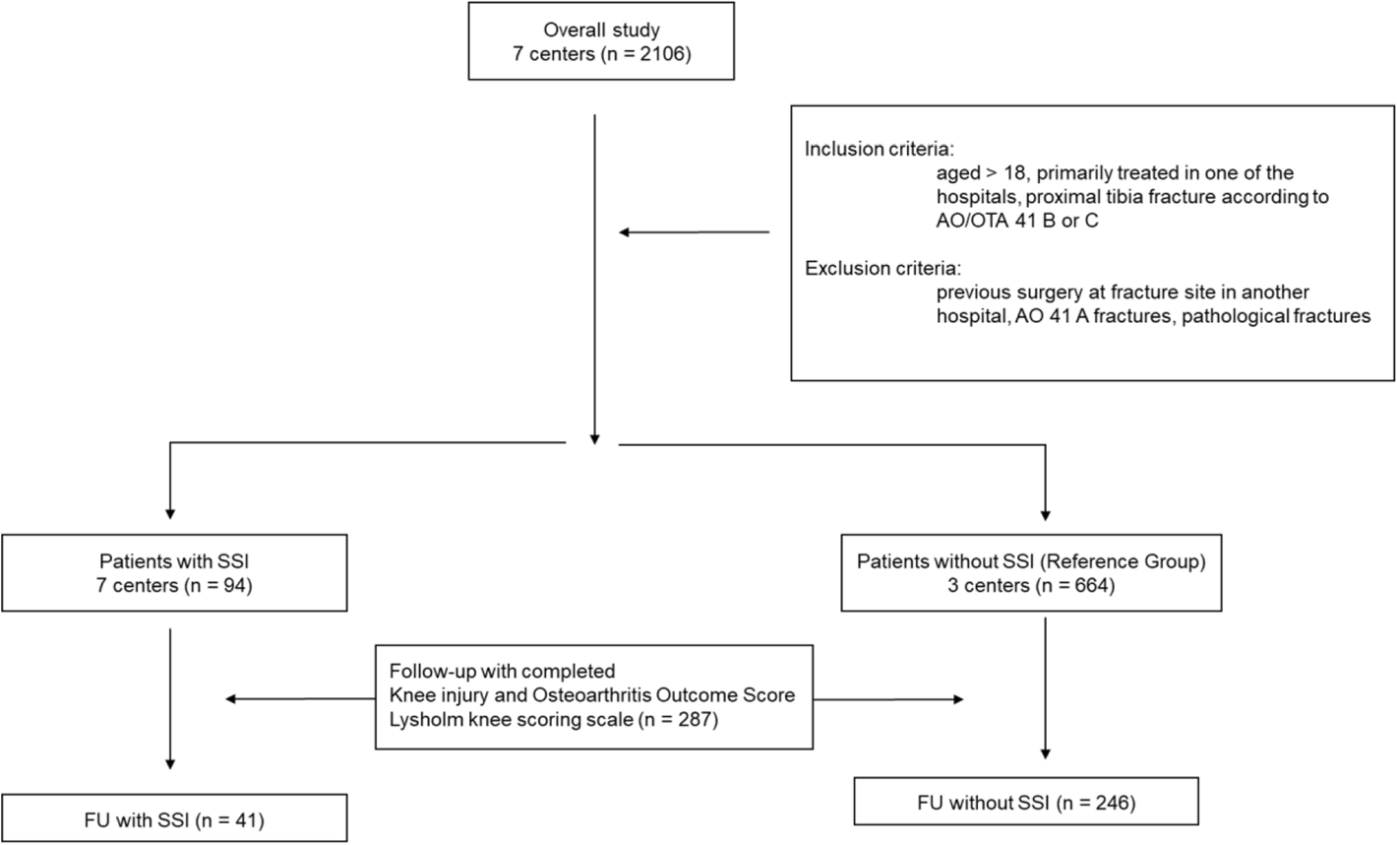
Flow diagram showing patient selection from different centers. FU, follow-up; SSI, surgical site infection; AO, Arbeitsgemeinschaft für Osteosynthesefragen Foundation; OTA, Orthopaedic Trauma Association

Of those 94 patients, 41 could be evaluated according to the study protocol (43.6 %). As the reference group, 246 patients without SSI with follow-up data were included from three centers (37.0 %). In summary, 287 patients with an average follow-up of 75.9 ± 35.9 months (range, 14–146 months) were included in this study (Fig. 1). Patients with SSI had a mean follow-up of 70.7 ± 39.0 months (range, 14–140 months), and patients without SSI had a mean follow-up of 78.9 ± 35.4 months (range, 20–146 months), with no significant difference between the groups (*p* = 0.3).

### Descriptive data

Basic demographic data for the collective and the two subgroups (patients with SSI and those without SSI) are presented in Tables 1 and 2. Patients in the overall group were on average 50.7 years of age, had a body mass index (BMI) of 26.8 kg/m^2^, and a percentage of women of 49.1 %. There was no significant difference between the groups in terms of age, the presence of diabetes mellitus, BMI, and comorbidities. However, there was a significant difference in body weight, sex, and smoking and drug abuse between the groups (Table 1).

**Table 1:** Descriptive data of the overall population and the subgroups with and without SSI

		SSI group (*n* = 41)		Non-SSI group (*n* = 246)		Total (*n* = 287)		*p*
		**Mean**	**SD**	**Mean**	**SD**	**Mean**	**SD**	
Age	y	52.1	11.1	50.5	14.2	50.7	13.8	0.4
Weight	kg	85.0	18.2	78.1	18.4	79.2	18.5	**0.03**
BMI	kg/m²	27.7	5.1	26.7	5.4	26.8	5.4	0.2
		**n**	**%**	**n**	**%**	**n**	**%**	
Sex	Male	28	68.3 %	113	45.9 %	141	49.1 %	**0.01**
	Female	13	31.7 %	133	54.1 %	146	50.9 %	
Comorbidities	None	20	48.8 %	136	55.3 %	156	54.4 %	
	1–3	17	41.5 %	95	38.6 %	112	39.0 %	
	4–5	3	7.3 %	4	1.6 %	7	2.4 %	
	≥ 6	1	2.4 %	7	2.9 %	8	2.8 %	0.2
Diabetes mellitus		5	12.2 %	16	6.6 %	21	7.3 %	0.3
Immunosuppression		1	2.4 %	3	1.2 %	4	1.4 %	
Smoking		16	39.0 %	50	20.3 %	66	23.0 %	**0.03**
Drug abuse		7	17.1 %	12	4.9 %	19	6.6 %	**0.01**

*BMI *body mass index, *SSI *surgical site infection, *SD *standard deviation

There was a significantly higher proportion of C fractures in the SSI group than in the non-SSI group. Additionally, the SSI group showed a significantly more complex fracture morphology, had significantly more patients with polytrauma or a relevant concomitant injury to the affected knee joint or other body region, and had significantly more open fractures or compartment syndrome than the non-SSI group (Table 2).

**Table 2:** Data of the overall population and subgroups with/without SSI concerning fracture morphology and concomitant injuries

		SSI group (*n* = 41)		Non-SSI group (*n* = 246)		Total (*n* = 287)		*p*
		**n**	**%**	**n**	**%**	**n**	**%**	
AO category	B	8	19.5	142	57.7	150	52.3	**< 0.001**
	C	33	80.5	104	42.3	137	47.7	
AO subcategory								
	B1	2	4.8	24	9.8	26	9.1	
	B2	1	2.4	44	18.0	45	15.7	
	B3	5	12.2	74	30.3	79	27.5	
	C1	3	7.3	18	7.4	21	7.3	
	C2	6	14.6	12	4.9	18	6.3	
	C3	24	58.5	74	30.3	98	34.1	
Concomitant injury of the affected knee		22	53.6	96	36.4	118	41.1	**0.01**
Compartment syndrome		16	39.0	14	5.7	30	10.5	**< 0.001**
Open fracture		9	22.0	7	2.9	16	5.6	**< 0.001**
Concomitant injury of other body region		20	48.8	47	19.3	67	23.3	**< 0.001**
Polytrauma		8	19.5	17	7.0	25	8.7	**0.01**

*SSI *surgical site infection

Regarding surgical treatment, an external fixator was applied significantly more frequently in the SSI group than in the non-SSI group. A list of the primary surgical procedures in both groups and in the overall population is shown in Table 3.

**Table 3:** Surgical procedures of the whole population and the subgroups with and without SSI

Surgical procedure	SSI group (*n* = 41)		Non-SSI group (*n* = 246)		Total (*n* = 287)	
	**n**	**%**	**n**	**%**	**n**	**%**
External fixation	26	63.4	55	22.6	81	28.2
Plate	8	19.5	69	28.3	77	26.8
Screw	1	2.4	28	11.5	29	10.1
Plate and screw	3	7.2	75	30.8	78	27.2
Double plate	3	7.2	14	5.7	17	5.9
TKA	0	0.0	2	08	2	0.7
Other	0	0.0	3	1.2	3	1.0

*TKA* total knee arthroplasty, *SSI* surgical site infection

### Outcome data

**Fig. 2:**
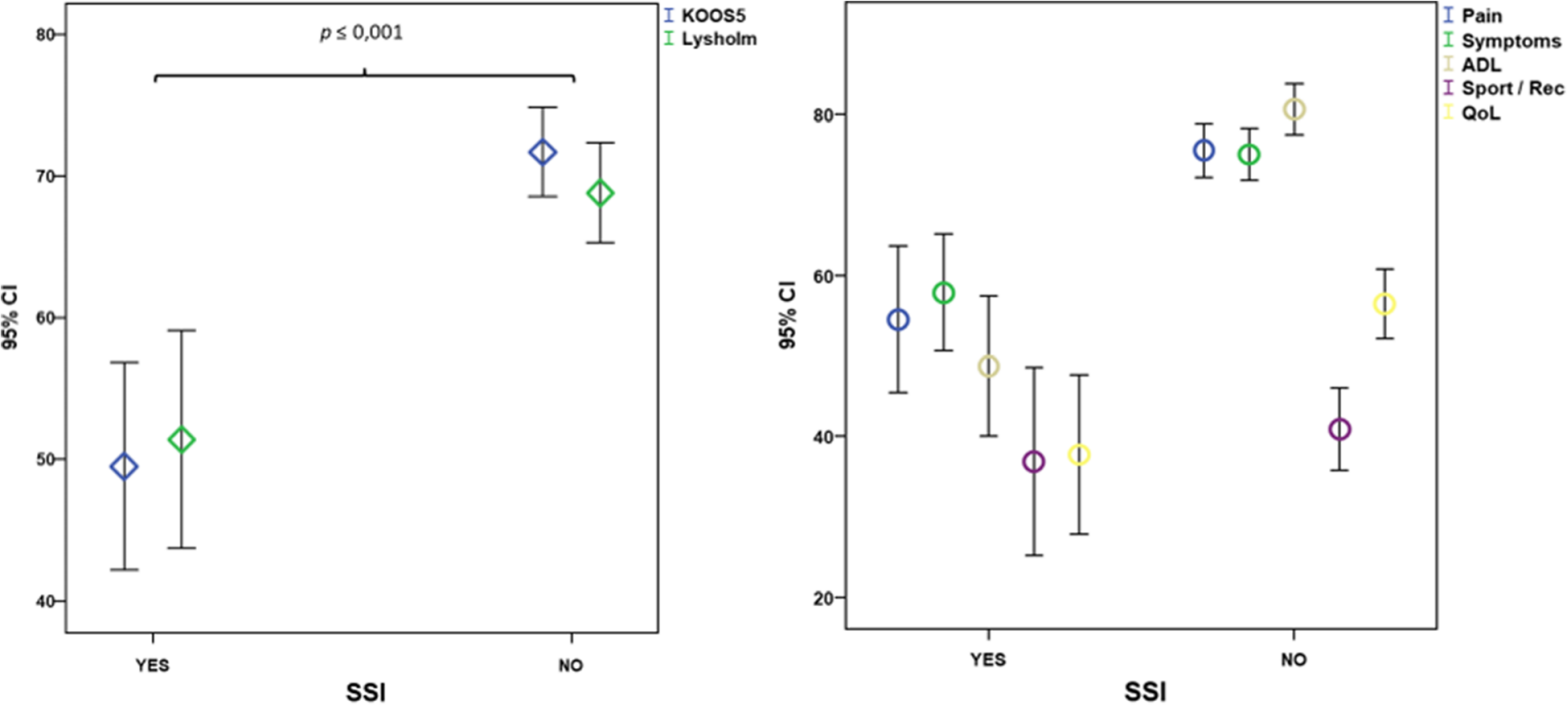
Comparison of the KOOS5, Lysholm knee scoring scale score, and KOOS subscores in patients with and without SSI. ADL, activities of daily living; Sport/Rec, function/sports and recreational activities; QoL, quality of life; CI, confidence interval; KOOS, Knee injury and Osteoarthritis Outcome Score; KOOS5, overall Knee injury and Osteoarthritis Outcome Score

Compared to patients without SSI, those with SSI had a significantly poorer outcome based on the KOOS5 score (48.7 ± 23.2 versus [vs.] 71.5 ± 23.5) and Lysholm knee scoring scale score (51.4 ± 24.0 vs. 71.4 ± 23.5) (Fig. 2; Table 4). Significant differences between the SSI and non-SSI groups were also evident in the KOOS subscores for pain (57.9 ± 22.9 vs. 75.0 ± 22.3), symptoms (54.5 ± 28.8 vs. 75.4 ± 23.4), ADL (48.8 ± 27.5 vs. 80.5 ± 22.6), and QOL (37.8 ± 31.5 vs. 56.4 ± 30.2). For Sports/Rec, the KOOS5 subscore was not significantly different between the groups (36.9 ± 37.1 vs. 41.0 ± 35.7) (Fig. 2; Table 4). Furthermore, the Lysholm knee scoring scale score differed significantly between the groups (51.4 ± 24.0 vs. 71.4 ± 23.5, *p* = 0.001).

**Table 4:** Outcome based on the KOOS5 subscores and Lysholm knee scoring scale scores

	SSI group (*n* = 41)				Non-SSI group (*n* = 246)							
	Mean	SD	Min	Max	Mean	SD	Min	Max	Diff	95 % confidence interval		*p*
KOOS symptoms	57.9	22.9	14.3	100	75.0	22.3	10.7	100	-17.1	-25.0	-9.3	**< 0.001**
KOOS pain	54.5	28.8	0.0	100	75.4	23.4	2.8	100	-20.9	-30.5	-11.2	**< 0.001**
KOOS ADL	48.8	27.5	0.0	100	80.5	22.6	0.0	100	-31.7	-40.9	-22.5	**< 0.001**
KOOS Sport/Rec	36.9	37.1	0.0	100	41.0	35.7	0.0	100	-4.2	-16.9	8.5	0.51
KOOS QoL	37.8	31.5	0.0	100	56.4	30.2	0.00	100	-18.7	-29.4	-7.9	**0.001**
KOOS5	48.7	23.2	5.4	97.6	71.5	21.8	7.00	100	-22.8	-30.7	-14.9	**< 0.001**

*SD* standard deviation, *ADL* activities of daily living, *Sport/Rec* function/sports and recreational activities, *QoL* quality of life, *min* minimum, *max* maximum, *diff* difference, *KOOS* Knee injury and Osteoarthritis Outcome Score, *KOOS5* overall Knee injury and Osteoarthritis Outcome Score

### Multivariable models

SSI remained an important risk factor in the multivariable models after adjustment for possible confounders. The models estimated clinically relevant mean decreases of the KOOS: 16 for the sum score, 15 for the symptoms subscore, 13 for the pain subscore, and 23 for the ADL subscore. Similarly, SSI was associated with about a 10-point decrease in the Lysholm knee scoring scale score. The effects of SSI on the Sport/Rec subscore (3.5 points) and QoL subscore (6.5 points) were much lower that those on the other subscores (Table 5). The lack of SSI in the “best models” for these scales indicates that other covariates are much stronger associated with them.

Nearly all scales were strongly associated with the number of pre-existing conditions with effects up to 30 points. Similarly, AO fracture class C was associated with a mean decrease of about 10 points. Compartment syndrome was associated with the sum score, pain subscore, ADL subscore, and QoL subscore (about a 10-point decrease too). The effect of concomitant injury in some models was small. Body weight (estimated 2–2.5 points per 10kg) was weakly associated with the KOOS subscore for QoL and the Lysholm knee scoring scale score. Finally, an increase by 5 points of the KOOS subscore for pain was associated with external fixation, but this was not significant (Table 5).

**Table 5:** Data of multivariate analysis after adjusting for possible confounders

		Coefficient	95 % Confidence interval			*p*	
KOOS	SSI	-15.3	-23.3	-7.2	< 0.001		
symptoms	AO C vs. B	-11.2	-17.3	-5.1	< 0.001		
	Concomitant injury	-5.9	-10.0	-1.8	0.004		
KOOS	SSI	-13.3	-22.8	-3.7	0.006		
pain	Pre-existing conditions						
	1–3	-4.0	-10.4	2.4	0.22		
	4–5	-11.3	-30.0	7.4	0.23		
	≥ 6	-27.6	-51.8	-3.4	0.023		
	AO C vs. B	-12.1	-19.6	-4.5	0.002		
	Compartment syndrome	-10.9	-22.8	1.1	0.070		
	External fixation	5.1	-3.7	13.9	0.25		
KOOS	SSI	-22.8	-31.6	-14.0	< 0.0.001		
ADL	Pre-existing conditions						
	1–3	-9.1	-15.1	-3.0	0.003		
	4–5	-12.3	-29.9	5.3	0.165		
	≥ 6	-30.3	-53.1	-7.6	0.008		
	AO C vs. B	-10.5	-17.0	-4.0	0.001		
	Compartment syndrome	-10.6	-21.7	0.5	0.058		
KOOS	Pre-existing conditions						
Sport/Rec	1–3	-20.7	-29.9	-11.4	< 0.001		
	4–5	-23.7	-50.3	2.9	0.076		
	≥ 6	-23.7	-58.5	11.1	0.175		
	AO C vs. B	-9.8	-19.0	-0.5	0.036		
	Concomitant injury	-6.8	-13.3	-0.3	0.036		
KOOS	Body weight (10 kg)	-2.5	-4.8	-0.2	0.033		
QoL	Pre-existing conditions						
	1–3	-8.3	-16.2	-0.4	0.037		
	4–5	-19.1	-41.9	3.6	0.094		
	≥ 6	-14.2	-43.9	15.6	0.34		
	AO C vs. B	-14.2	-22.6	-5.8	< 0.001		
	Compartment syndrome	-15.9	-29.6	-2.2	0.021		
KOOS5	SSI	-16.2	-24.7	-7.6	< 0.001		
	Pre-existing conditions						
	1–3	-8.4	-14.1	-2.7	0.004		
	4–5	-13.7	-30.2	2.8	0.099		
	≥ 6	-23.5	-44.8	-2.1	0.029		
	AO C vs. B	-10.7	-16.8	-4.5	0.001		
	Concomitant injury	-3.6	-7.7	0.5	0.082		
	Compartment syndrome	-9.1	-19.6	1.3	0.081		
Lysholm knee scoring scale	SSI	-11.4	-22.1	-0.6	0.036		
	Pre-existing conditions						
	1–3	-7.9	-15.8	0.0	0.047		
	4–5	-14.1	-37.0	8.7	0.22		
	≥ 6	-17.5	-47.3	12.3	0.24		
	AO C vs. B	-15.3	-23.5	-7.0	< 0.001		

*ADL* activities of daily living, *Sport/Rec* function/sports and recreational activities, *QoL* quality of life, *SSI* surgical site infection, *KOOS* Knee injury and Osteoarthritis Outcome Score, *KOOS5* overall Knee injury and Osteoarthritis Outcome Score, *KOOS* Knee injury and Osteoarthritis Outcome Score, *KOOS5* overall Knee injury and Osteoarthritis Outcome Score, *AO* Arbeitsgemeinschaft für Osteosynthesefragen Foundation

## Discussion

In terms of PROs, patients with SSI had a significantly poorer outcome than those without SSI. Even after adjusting for possible confounders, SSI remained an important factor in multivariable models for poor outcome. Previously, it was postulated that regardless of the therapeutic approach, relevant impairments in subjective function of the knee joint are common and are explained by the severity of intraarticular bicondylar fractures with multifragmented parts in combination with injury to the cartilage and intraarticular soft tissue structures [26]. For this reason, in addition to SSI, other important factors that can cause a poor outcome should be considered.

After comparing our results with findings from currently available studies that have used the KOOS and Lysholm knee scoring scale as PROs, it is evident that patients with SSI have an even worse outcome based on the KOOS or Lysholm knee scoring scale score. This is independent of whether a subgroup with poor outcome was defined in these studies due to malalignment, insufficient reduction of the fracture, or any serious complication other than SSI [2, 3, 5–9, 17–19]. For example, Singleton et al. showed that the KOOS worsens depending on the articular congruity after reduction [5]. However, patients with SSI had a significantly worse outcome (KOOS pain subscore, 54.5; KOOS ADL subscore, 48.8; KOOS QoL subscore, 37.8) than the subgroup of patients with > 5 mm of articular depression (KOOS pain subscore, 69.4; KOOS ADL subscore, 78.1; KOOS QoL subscore, 52.8) in this study. Furthermore, Jansen et al. evaluated 23 AO C fractures (follow-up duration, 67 months) with an overall KOOS of 67.84 and Lysholm knee scoring scale score of 66.2; the subgroup with postoperative malalignment had a better outcome (KOOS 58 ± 14) than the SSI group in our study (KOOS5, 48.7 ± 23.2; Lysholm knee scoring scale score, 51.4 ± 24.0) [7]. Larsen et al. included complex bicondylar TPFs (AO/OTA 41 C) in their study, and the mean KOOS subscores were as follows: pain, 72.5; symptoms, 62.7; ADL, 75.9; Sport/Rec, 35.4; and QOL, 56.4. Their findings were comparable to our results overall, but their subscores were significantly better than those of our patients with SSI. [2] Additionally, in our study, multivariable analysis showed that AO fracture class C is associated with a mean decrease of 10 points. In addition to the fracture morphology, the influence of a relevant soft tissue injury in terms of compartment syndrome was shown. Thus far, different effects have been seen on this in other studies [27–29]. The number of previous diseases has a relevant influence on the KOOS subscore for ADL. Although the KOOS is a knee-specific score, the ADL questions refer to activities that that are no longer possible without restrictions in multimorbid patients (e.g., climbing stairs, going shopping, and walking on flat surface).

Finally, our data showed that SSI is responsible for a poor outcome after surgical treatment of a TPF regardless of other risks or influencing factors. These strong results show that in addition to optimal fracture reduction and postoperative management, the avoidance of SSI has the greatest impact on a good outcome in patients with TPF.

### Limitations

The strongest limitation of our study was the retrospective study design. Epidemiological data were complete from all participating centers, but only a small number of patients could be reached for follow-up (43.6 and 37.0 %). Because of this small number of responders, potential bias cannot be excluded. However, in terms of epidemiological data, there was no significant difference between the groups with and without SSI regardless of whether follow-up data were available.

## Conclusions

Compared to previous studies, validated PRO scores demonstrated that the impact of SSI in patients with surgically treated TPFs is dramatic, in terms of not only pain and symptoms but also ADL and QoL, compared to that in patients without SSI.

## Data Availability

The datasets used and/or analyzed during the current study are available from the corresponding author on reasonable request.

## Abbreviations

ADL: Activities of daily living
AO/OTA: Arbeitsgemeinschaft für Osteosynthesefragen Foundation/Orthopaedic Trauma Association
BMI: Body mass index
KOOS: Knee injury and Osteoarthritis Outcome Score
KOOS5: Overall Knee injury and Osteoarthritis Outcome Score
ISS: Injury severity score
PRO: Patient-reported outcome
QoL: Quality of life
SD: Standard deviation
Sport/Rec: Sports and recreation
SSI: Surgical site infection
TPF: Tibial plateau fracture
